# Influence of fermentation temperature on in situ heteropolysaccharide formation (*Lactobacillus plantarum* TMW 1.1478) and texture properties of raw sausages

**DOI:** 10.1002/fsn3.2054

**Published:** 2021-01-23

**Authors:** Lina Velasco, Myriam Loeffler, Isabel Torres, Jochen Weiss

**Affiliations:** ^1^ Department of Food Material Science Institute of Food Science and Biotechnology University of Hohenheim Stuttgart Germany

**Keywords:** fermentation temperature, heteropolysaccharides, salami, starter culture, texture

## Abstract

This study puts a focus on the influence of microbial in situ heteropolysaccharide (HePS) formation on the quality of raw fermented sausages (salami). Since exopolysaccharide‐production is often triggered by sub‐optimal growth conditions, the influence of different fermentation temperatures was also investigated. For this reason, the sausage batter was inoculated with (*Lactobacillus plantarum* TMW 1.1478) or without (*L. sakei* TMW 1.2037; control) a HePS‐producing starter culture (inoculation concentration ~10^8^ CFU/g), and the sausages fermented at either 10, 16, or 24°C (7 days), followed by a drying period at 14°C until the final weight loss of 31% was reached. Microbial growth, pH, and weight loss development were monitored and the products further characterized using texture profile analysis and a sensory test. HePS in the salami matrix were determined using confocal laser scanning microscopy and a semi‐quantitative data interpretation approach. Sausages containing *L. plantarum* were found to be significantly (*p* < .05) softer compared with control samples, which was also confirmed in the sensory analysis. The different fermentation temperatures had an influence on the drying speed. Here, sausages produced with *L. plantarum* needed more time to reach the final weight loss of 31% as compared to control samples, which could be attributed to the presence of exopolysaccharides in the matrix (*p* < .05). Using HePS‐forming starter cultures in raw fermented sausage manufacturing can lead to products with a softer texture (undesired in Europe) depending on the strain and processing conditions used, highlighting the importance of a suitable starter culture selection in food processing.

## Graphical abstract

This study deals with the influence of microbial in situ heteropolysaccharide (HePS) formation on the quality of raw fermented sausages (salami). HePS production was studied under different fermentation conditions (10–24°C) and products compared to salamis that have been produced with a non‐EPS forming starter culture. Sausages containing *L. plantarum* TMW 1.1478 were found to be significantly (*p* < .05) softer compared to control samples, which was also confirmed in the sensory analysis.

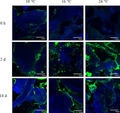

## 1 INTRODUCTION

Several exopolysaccharide (EPS)‐producing lactic acid bacteria are used in food production due to EPS having texturizing, viscosifying, gelling, and emulsifying properties (Lynch et al., 2018). Moreover, in situ produced EPS do not need to be labeled on the package, accounting for the consumers demand for more “natural” products. According to the mechanism of biosynthesis and composition, EPS can be classified into heteropolysaccharides (HePS) and homopolysaccharides (HoPS) (de Vuyst & Degeest, 1999). HoPS such as dextran or levan consist of a single type of monosaccharide and are extracellularly synthesized from sucrose or starch, whereas HePS are usually composed of repeating monosaccharide units ranging in size from disaccharides to octasaccharides and are synthesized in a complex, energy‐demanding biosynthesis from different sugars (Donot et al., 2012; Monsan et al., 2001). The ratio of the monosaccharides, the degree of branching as well as the linkage type and presence of other substituents affect both the molecular weight as well as the overall charge of the HePS, all influencing physicochemical interactions with food ingredients. This is also the reason why usually lower amounts of HePS are required to cause structural and to some extend textural changes in food products when compared to HoPS (Wingender et al., 2012). Previous studies showed that EPS‐production from mostly mesophilic bacteria can be improved under sub‐optimal growth conditions due to, for example, environmental stress (Bengoa et al., 2018; Prechtl et al., 2018a). In case of raw fermented sausage production (*salami*), nitrite curing salt may for instance favor microbial EPS formation by lactic acid bacteria, which are traditionally used in salami production to control microbial safety and sensory properties (Cocolin et al., 2011; Leroy et al., 2006). While a lot of research has been done with regard to EPS formation in dairy or bakery products (Abedfar et al., 2019; Bachtarzi et al., 2019; Gemelas et al., 2018; Yilmaz et al., 2015), very few studies focused on the usage of EPS‐forming starter cultures in meat products although they have been associated with health benefits besides their technofunctional properties. The meat industry is, however, constantly seeking for new starter cultures possessing different fermentation kinetics or other metabolic activities of interest. According to Dertli et al. (2016) who examined the influence of HePS‐producing *L. plantarum* 162 R and *Leuconostoc mesenteroides* N6 on the physicochemical, microbiological, microstructural, and textural properties of lamb and beef‐based Turkish sucuk, products containing the EPS‐producing strains were harder and less adhesive as compared to control samples. In contrast to that Hilbig, Gisder, et al. (2019) reported on fat‐reduced teewurst with an improved spreadability when EPS‐forming strains (HoPS: *Lactobacillus sakei* TMW 1.411 or HePS: *L. plantarum* TMW 1.1478) had been used during production. Latter effect would be crucial when it comes to dry fermented sausage production since softer products are usually associated with a less good quality (especially in European countries). These results highlight the importance of an EPS screening under fermentation conditions and the need for a better understanding of microbial in situ EPS formation in meat matrices and their influence on product properties. The present study therefore aims to (a) get a better understanding of the influence of HePS‐production on quality attributes of raw fermented sausages (*salami*) and to (b) investigate the influence of varying fermentation temperatures as an additional stress besides the present salt content, which may lead to an improved HePS‐production. For this reason, three fermentation temperatures have been investigated, two that are traditionally used in raw fermented sausage production (24°C and 16°C *in variable temperature fermentation*) and one that is far below the optimal fermentation temperature (10°C) accounting for an EPS formation under enhanced stress conditions.

## 2 MATERIALS AND METHODS

### 2.1 Materials

#### 2.1.1 Ingredients for raw sausage production

Pork meat and pork fat were purchased from a local wholesaler (MEGA eG) and standardized according to the GEHA meat classification system (Prändl et al., 1988) to S II and S VIII, respectively. Nitrite curing salt (NCS, 0.5% nitrite) was provided by Südsalz GmbH, ascorbic acid, and black pepper were purchased from Gewürzmüller, and dextrose as well sucrose was obtained from Südzucker AG.

#### 2.1.2 Microbiological culture medium and chemicals

De Man, Rogosa, and Sharpe (MRS) agar and broth (peptone from casein 10.0 g/L, meat extract 10.0 g/L, yeast extract 4.0 g/L; D (+)‐glucose 20.0 g/L, dipotassium hydrogen phosphate 2.0 g/L, Tween^®^ 80 1.0 g/L, di‐ammonium hydrogen citrate 2.0 g/L, sodium acetate 5.0 g/L, magnesium sulfate 0.2 g/L, and manganese sulfate 0.04 g/L, with [agar] or without [broth] agar‐agar 14.0 g/L), as well as Anaerocult^®^ were purchased from Merck KGaA. Peptone water (pH 7.0 ± 0.2; 5 g/L) was purchased from Carl Roth GmbH & Co. KG. Plate Count Agar (PCA; agar 15.0 g/L, glucose 1.0 g/L, peptones 5.0 g/L, and yeast extract 2.5 g/L) was obtained from AppliChem GmbH. Anaerocult^®^ was used to assure an anaerobic atmosphere during the incubation of MRS agar. Preparation: 35 mL of water was distributed over 1 sachet of Anaerocult. Calcofluor White Stain, Concanavalin A, and Glycerol (≥99.0%) were purchased from Sigma‐Aldrich Chemie GmbH. All microbiological media were prepared as specified by the respective manufacturers and autoclaved for 15 min at 121°C.

### 2.2 Methods

#### 2.2.1 Starter culture preparation

*Lactobacillus plantarum* TMW 1.1478 (HePS‐forming strain; henceforth referred to as *L. plantarum* 1.1478), and *Lactobacillus sakei* TMW 1.2037 (non‐EPS‐forming control strain; henceforth referred to as *L. sakei* 1.2037) were obtained from the Technical University of Munich (Department of Technical Microbiology). Bacterial strains were stored in MRS broth containing 20% (v/v) glycerol at −80°C. Before being used to inoculate the raw sausage mass, both *Lactobacillus* strains were activated in MRS broth for 48 hr at 30°C. To obtain a higher concentration of the initial inoculums (target: 10^8^ CFU/g meat), the solutions were centrifuged (Universalzentrifuge Hermle Z32HK, Hermle Labortechnik GmbH) at 2,352 *g* for 10 min at 25°C and the pellets then suspended by using small amounts of peptone water (c = 5 g/L). This had the additional benefit that the taste of the final products was not influenced by the presence of MRS broth.

#### 2.2.2 Raw fermented sausage production

The production of salami was performed in the pilot plant of the University of Hohenheim following a standard raw fermented sausage formulation (35% minced pork shoulder S II, 45% frozen pork meat S II, and 20% frozen pork back fat S VIII). After mincing the pork shoulder in a meat grinder with a 3 mm whole plate (Type Wd 114, Seydelmann), it was mixed with the remaining pork meat, back fat, and spices (3.0 g/kg black pepper, 5.0 g/kg sucrose, and 0.5 g/kg ascorbic acid) using a vacuum bowl chopper (Type K64 DC, Seydelmann). In total 64 kg of sausage, batter was produced and divided into two batches. The first batch was inoculated with the non‐EPS‐forming strain *L. sakei* 1.2037 (control; ~10^8^ CFU/g) and the second batch with the HePS‐producing strain *L. plantarum* 1.1478 (~10^8^ CFU/g). Same quantities but different raw materials (to e.g., account for differences in the raw material quality) were used to perform the repetition of the experiment. After adding and distributing the starter cultures, 28.0 g/kg nitrite curing salt (NCS) was added (short mixing step to homogenously distribute the salt) and the sausage batter then filled (MWF 591, MADO Patron) into casings (50 mm diameter; Nalo fasser S, Werner Niedenbeger GmbH). Sausages containing the control strain and those containing *L. plantarum* 1.1478 were further divided into three portions to allow for exposure to three different fermentation temperatures (10, 16, or 24°C) during the next 7 days, followed by a ripening (drying) period at 14°C until all sausages reached 31% of weight loss. Additionally, sausages were smoked after 24, 48, and 72 hr of fermentation in a smoking chamber at 24°C for 15 min.

#### 2.2.3 Microbiological analysis of raw fermented sausages

Microbiological analysis of raw fermented sausages was conducted at time 0 (raw material ± starter culture), after 24, 48, and 72 hr, and after 9, 13, and 15 days of production. To obtain aerobic and anaerobic viable cell counts of the different products, always 10 g of sample was taken aseptically from the core of the respective salami, transferred to a sterile filter bag and subsequently mixed for 1 min (6 strokes/second) with 90 mL of buffered peptone water (5 g/L) using a Masticator (Laborhomogenisator, IUL Instrument GmbH). Appropriate dilutions were plated on plate count agar (PCA; raw material quality) and on deMan, Rogosa, and Sharpe (MRS) agar using an automated spiral plater (Don Whitley Scientific) followed by incubation at 30°C for 24–48 hr under either anaerobic (MRS) or aerobic (PCA) conditions. Subsequently, the colonies were counted using an automatic colony counter (Acolyte, Synbiosis). Two independent samples were analyzed in triplicate.

#### 2.2.4 pH measurement & water activity

The pH values were monitored during fermentation and drying using a pH meter (WTW Microprocessor pH Meter, WTW GmbH), whereas the water activity was determined using an “Aqua Lab” device (CX‐2‐ Decagon Devices Inc.). Two independent samples were analyzed in triplicate.

#### 2.2.5 Weight loss measurement

The weight loss (target: 31%) of the sausages was gravimetrically monitored and calculated according to Equation 1:

(1) $$\text{WT}\;\text{loss}\;\left[\%\right]\;=\;\frac{M_{\text{initial}}\;‐\;M_{\text{end}}}{M_{\text{initial}}}\;\times \;100$$

where *M*_initial_ is the weight of the sample prior to the fermentation process (time 0; after filling) and *M*_end_ is the recorded weight after a specific processing time.

#### 2.2.6 EPS examination and quantification using confocal laser scanning microscopy (CLSM)

A qualitative assessment of the EPS‐production was performed after 0, 24, 48, 72 hr, and during the drying phase using the method as provided by Hilbig et al. (2019a), which is based on a method developed by Hassan et al. (2002). A cylindrical metal pipe was used to take a sample (0.5 cm high, 1.5 cm wide) from the core of the respective raw fermented sausage sample, which was then stained with 10 µL of a diluted (1:20) Concanavalin A solution (stock: 5 mg lyophilized powder in 5 mL phosphate buffer 10 mmol; pH 6) to determine in situ formed EPS. Proteins were made visible by adding Calcoflour White Stain (10 µL) after 60 min of dark incubation at 12°C. The samples were analyzed using a Nikon Eclipse‐Ti Inverse Microscope D‐ Eclipse C1 (Nikon GmbH) and a 60‐fold magnification lens with immersion oil. A red helium‐neon laser at 638 nm and an argon laser at 488 nm were used for the excitation of EPS and proteins. At least nine pictures of each sample were taken and further analyzed. Scales were inserted using the ImageJ software (version 1.4.3.67, National Institutes of Health) after creating a RGB picture using the EZ‐C1 3.70 Imaging software (Nikon GmbH). The semi‐quantitative analysis of formed EPS (expressed as green area) was performed using MATLAB (The Math Works, Inc., version R2013b 8.2.0.701) following a method developed by Bosse et al. (2015) who introduced the following equation (Equation 2), which was also used in the present study:

(2) $$\text{Green}\;\text{area}\;\text{picture}\;\left[\%\right]\;=\;\frac{\text{Green}\;\text{area}\;\left(\text{pixels}\right)}{\text{Total}\;\text{area}\;\text{pixcels}}\;\times \;100\%$$

#### 2.2.7 Texture profile analysis

A texture profile analysis (TPA) was performed as soon as the raw sausages achieved 21%, 26%, and 31% weight loss, respectively, to get deeper insights on the influence of EPS production during drying. Prior to performing the TPA, the sausages were equilibrated (12°C), sliced, and casings removed. Fifteen samples were taken from each batch (2 cm high × 1.5 cm wide; in filling direction) and analyzed using a double compression test (50%; 20 s interval between the two compression cycles) with a probe of 2.5 cm diameter, at a cross‐head speed of 50 mm/min using an Instron device (Model 1011, Instron Engineering Corp.) equipped with a 100 N load cell. The hardness of the samples was determined at the first peak of compression and the gumminess calculated by multiplying hardness with cohesiveness. The springiness index (deformation during the compression) and cohesiveness (stability of the sausage) were calculated according to Equations 3 and 4.

(3) $$\text{Springiness}\;\text{index}\;\left(‐\right)\;=\;\frac{\left(a\;‐\;b\right)}{\left(c\;‐\;d\right)}$$

with *a* being the distance to the maximal second compression (mm), *b* being the distance to the onset of the second compression (mm), *c* being the distance to the maximal first compression (mm), and *d* being the distance to the onset of the first compression (mm).

(4) $$\text{Cohesiveness}\;\left(‐\right)\;=\;\frac{\text{Second}\;\text{area}\;\left(J\right)}{\text{First}\;\text{area}\;\left(J\right)}$$

#### 2.2.8 Sensorial analysis

The sensory evaluation of the raw fermented sausages was performed with a panel of 20 untrained participants who were familiar with the product. Here, sausages containing either the control strain *L. sakei* 1.2037 or the HePS‐forming strain *L. plantarum* 1.1478 were cut into 1.5 cm thick slices with a diameter of about 3.5 cm and served at room temperature. For the evaluation, a paired comparison test was done with the control sample corresponding to a score of 5 (scale: 0–10). Samples containing the HePS‐forming strain had to be rated compared to the control regarding consistency, preference and taste with values ˂5 indicating a softer texture/consistency and a less good taste. The sensory test was carried out and recorded with the software Fizz Acquisition 2.51 and Fizz Acquisition 2.50 (both Biosystems).

#### 2.2.9 Statistical analysis

Each experiment was conducted twice and all measurements repeated three times. Means and standard deviations were calculated using Excel (Microsoft). SigmaPlot 12.5 (Systat software Inc.) was used to statistically evaluate the results using the Shapiro–Wilkes test for normality (*p* < .05; failed) and the Levene test for equal variance (*p* < .05). This was followed by a Kruskal–Wallis test and a multiple comparison procedure (Student–Newman–Keuls test) to determined significant differences (*p* < .05) between results gained from the TPA and EPS‐analysis, whereas a paired *t* test was performed to analyze differences between results gained from the sensory evaluation.

## 3 RESULTS AND DISCUSSION

### 3.1 Microbiological analysis of raw fermented sausage samples

**FIGURE 1:**
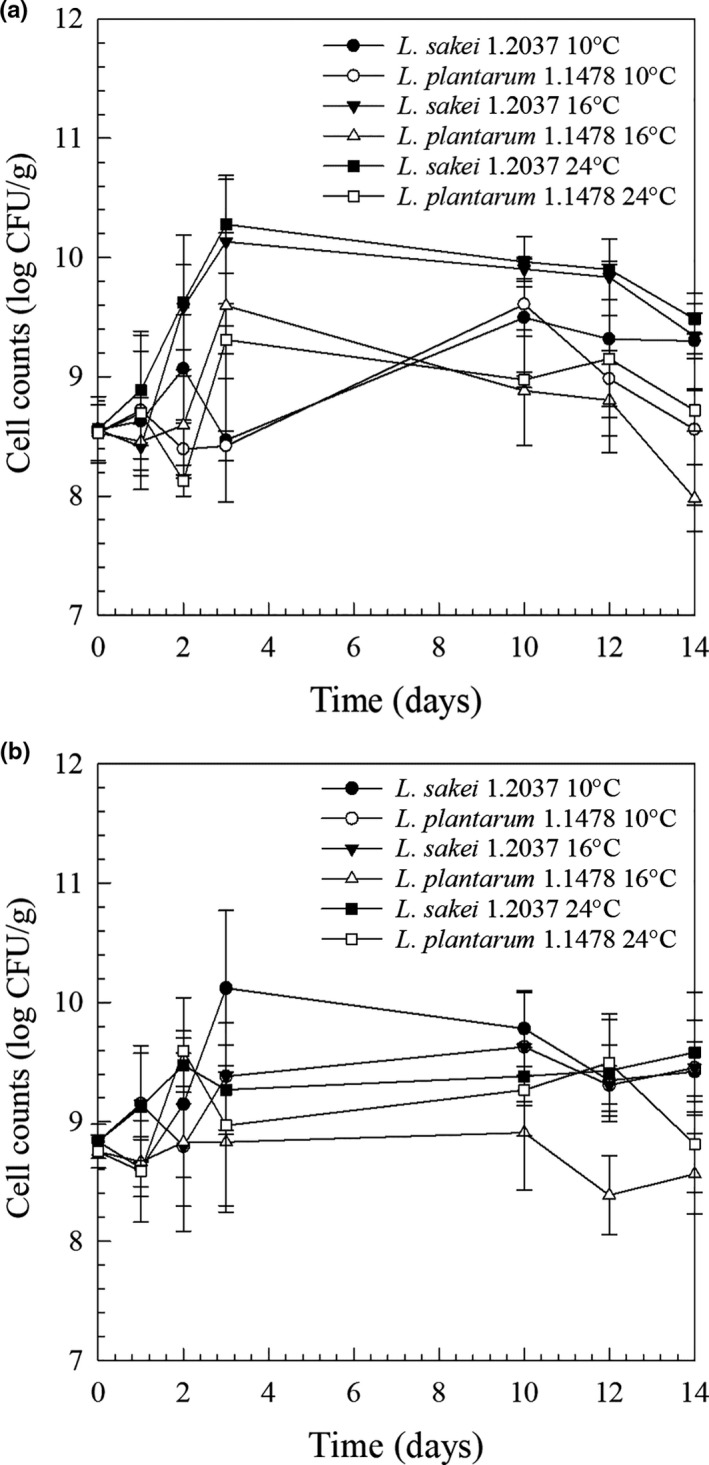
Anaerobic cell counts of raw fermented sausages that have been produced with the non‐EPS‐forming strain *L. sakei* TMW 1.2037 (control; ~10^8^ CFU/g) or the HePS‐forming strain *L. plantarum* TMW 1.1478 (~10^8^ CFU/g) of the first (a) and second experiment (b; independent sausage production). Measurements were carried out over the fermentation (performed at 10, 16, or 24°C) and drying phase until 31% weight loss was reached (~14 days)

The mesophilic aerobic viable cell counts (PCA) of the minced raw meat ranged between 5.70 × 10^3^ and 6.05 × 10^3^ CFU/g meat indicating a good raw material quality (Feiner, 2006). Among lactic acid bacteria known for HePS‐formation, *L. plantarum* has been widely studied in food mimicking model systems (Hilbig et al., 2019a; Prechtl et al., 2018b) as well as in bakery‐ (Abedfar et al., 2019), kefir‐ (Wang et al., 2015) and lately also in meat‐based products (Dertli et al., 2016; Hilbig et al., 2019b). Moreover, Hilbig, Gisder, et al. (2019) also used *L. plantarum* 1.1478 for fat‐reduced spreadable raw fermented sausage production. To some extent, this allows for a better data interpretation and presentation of mechanistic insights of the present study, even though the matrix structure of spreadable raw fermented sausages (*protein surrounded by fat*) is different from salami (*fat surrounded by protein*). To get a better understanding of the influence of HePS on product quality, sausages produced with the non‐EPS forming *L. sakei* 1.2037 were always compared to sausages containing *L. plantarum* 1.1478. Based on a previous study done by the authors, HePS‐production by *L. plantarum* 1.1478 in a salami matrix has been found to be more pronounced when using higher inoculation concentrations, which is why in the present study bacterial suspensions were concentrated prior to application leading to concentrations of almost 10^8^ CFU/g at time 0 hr (Figure 1). Taking the repetition (second independent production) and standard deviations into account, no clear influence of fermentation temperature (10, 16, or 24°C) on microbial growth behavior could be observed, which might be attributed to the inhomogeneous salami matrix with fat and potentially EPS acting as an additional barrier to colder temperatures (D'Amico et al., 2006). Moreover, due to inoculation concentrations being very high, no remarkable growth is expected but one can see a slight decrease of bacterial counts after 10 days of production.

### 3.2 pH, *a*_w_ and weight loss development in raw fermented sausages

**FIGURE 2:**
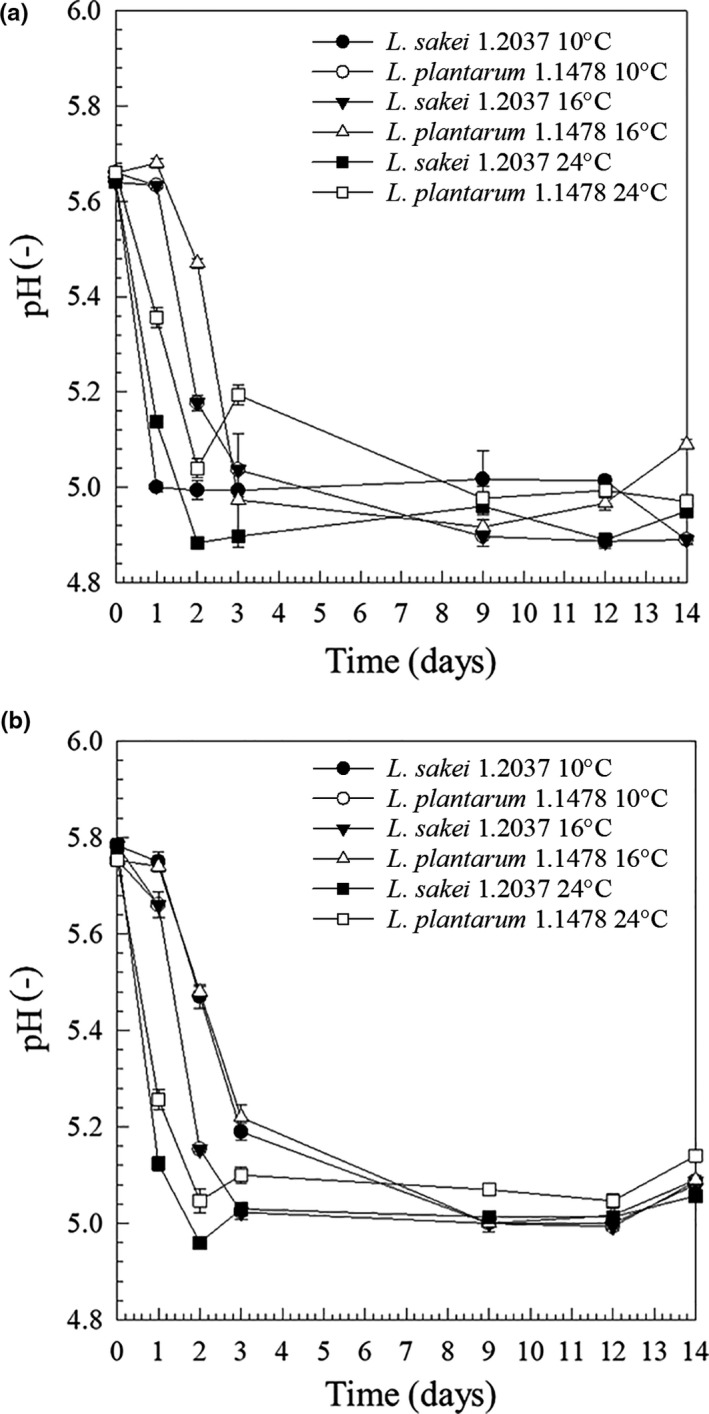
pH – values of the sausages produced with the non‐EPS‐forming strain *L. sakei* TMW 1.2037 (control; ~10^8^ CFU/g) or the HePS‐forming strain *L. plantarum* TMW 1.1478 (~10^8^ CFU/g) during the fermentation (performed at 10, 16, or 24°C) and drying phase of the sausages of the first (a) and second production (b; independent sausage production)

The optimal pH for EPS production can differ from the one associated with optimal growth, especially for strains showing a growth‐independent EPS formation (de Vuyst & Degeest, 1999; Looijesteijn et al., 1999) with a pH of 5.5–6.5 promoting EPS production of many *Lactobacillus strains* (Czaczyk & Myszka, 2007). Lactic acid bacteria such as *L. sakei* 1.2037 and *L. plantarum* 1.1478 are known to contribute to textural changes by acidifying the meat batter leading to a coagulation of muscle proteins (Laranjo et al., 2017). Changing fermentation conditions thus has an influence on textural properties. In the present study, same fermentation conditions have been used for both strains that showed similar fermentation kinetics with *L. plantarum* 1.1478 being slightly slower in the beginning which may be attributed to EPS metabolism. However, both strains led to products with pH values in the same range after storage. The values obtained are represented in Figure 2. Independent of the fermentation temperatures used, the pH values of raw sausages inoculated with the HePS‐forming strain *L. plantarum* decreased from 5.64 ± 0.01 to 4.89 ± 0.01, 5.09 ± 0.01, and 4.97 ± 0.01 at 10, 16, and 24°C, respectively. The pH of sausages that have been produced with the control strain *L. sakei* 1.2037 decreased from an initial pH value of 5.64 ± 0.01 to 4.89 ± 0.00 (10°C), 4.89 ± 0.01 (16°C), and 4.95 ± 0.01 (24°C), respectively, at the end of production (31% weight loss). The final values are in agreement with the typical pH range for salami products (pH 4.8–5.2). Similar results were obtained in the repetition (second independent production) with all values being slightly higher, including the pH right after production.

The final *a*_w_ values for raw fermented sausages from both independent productions were in the same range of 0.89 ± 0.01 and 0.88 ± 0.01, respectively.

Differences could, however, be seen with regard to the weight loss development of the products (Figure S1). Samples containing the control strain *L. sakei* 1.2037 reached the final weight loss of 31% after 14 days of storage, provided the initial fermentation temperature was set to 10 or 16 degrees, whereas it took only 11 days when the sausages were exposed to 24°C during the first 7 days of production. In contrast to that, inoculating sausages with the HePS‐forming strain *L. plantarum* 1.1478 (~10^8^ CFU/g) led to an increase of time needed to reach the final weight loss of 31% being 15, 14, and 13 days for products stored at 10, 16, and 24°C during the first 7 days of production, respectively. This can be a first indication that in situ HePS formation influences the properties of salami.

### 3.3 EPS detection, TPA, and sensory analysis

**FIGURE 3:**
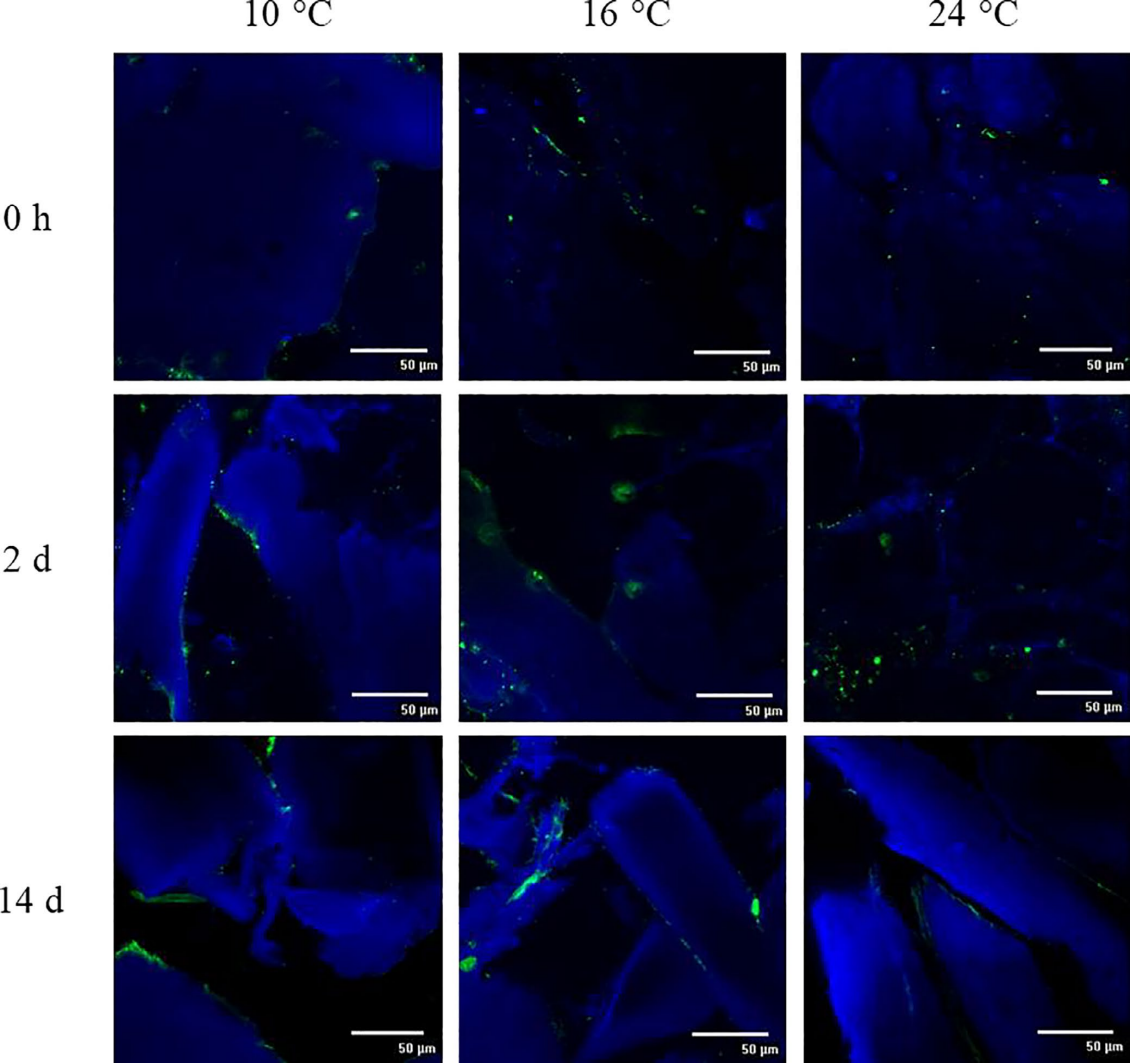
Qualitative EPS determination (CLSM, 600× magnification) in sausages produced with *L. sakei* TMW 1.2037 (control; ~10^8^ CFU/g) in dependency of the different fermentation temperatures used (10, 16, or 24°C). EPS are stained green and proteins are stained blue. The corresponding semi‐quantitative data (based on ≥9 pictures/sample) are presented in Table 1

**FIGURE 4:**
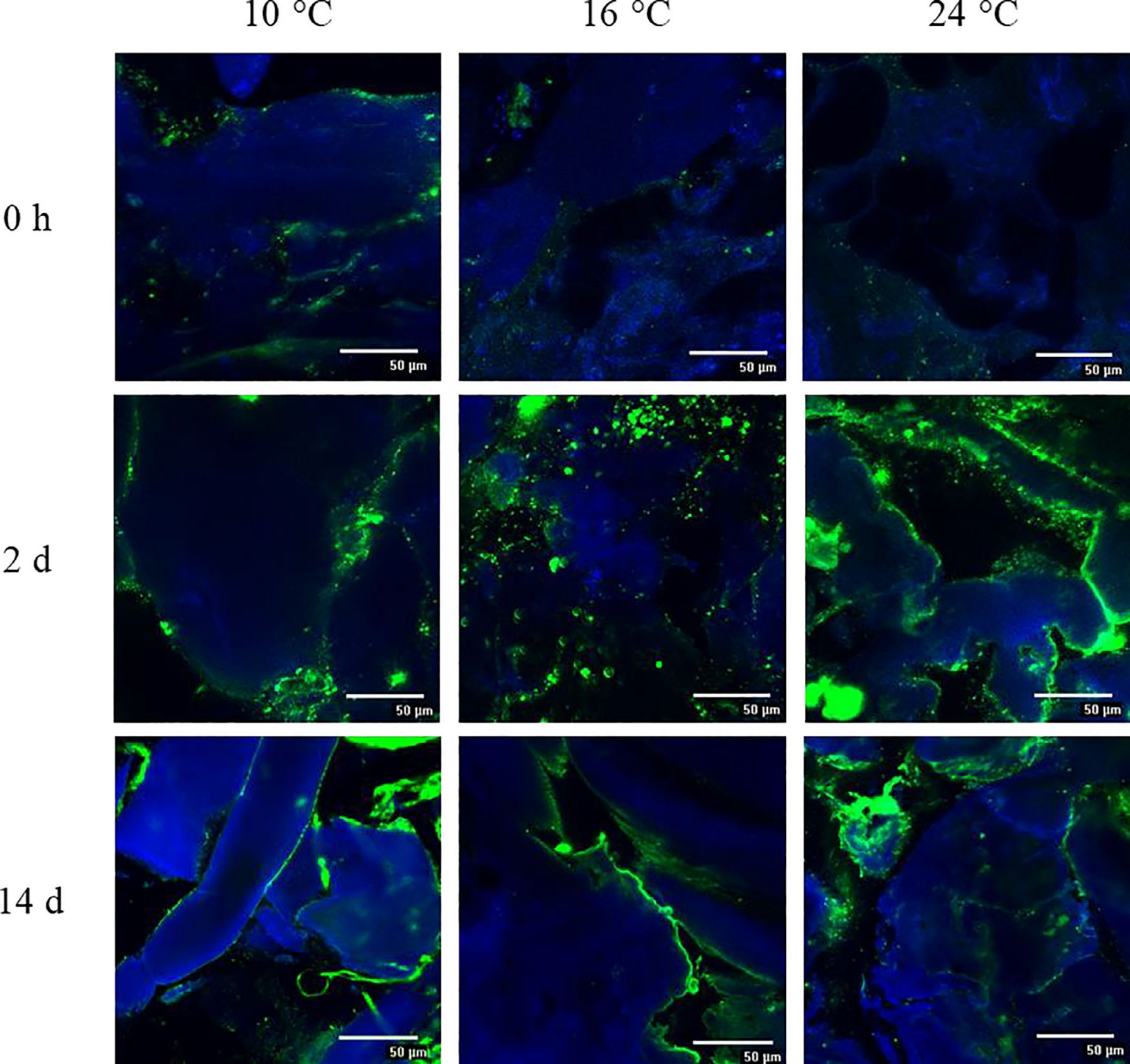
Qualitative EPS determination (CLSM, 600× magnification) in sausages produced with the HePS‐forming strain *L. plantarum* TMW 1.1478 (~10^8^ CFU/g) in dependency of the different fermentation temperatures used (10, 16, or 24°C). EPS are stained green and proteins are stained blue. The corresponding semi‐quantitative data (based on ≥9 pictures / sample) are presented in Table 1

**FIGURE 5:**
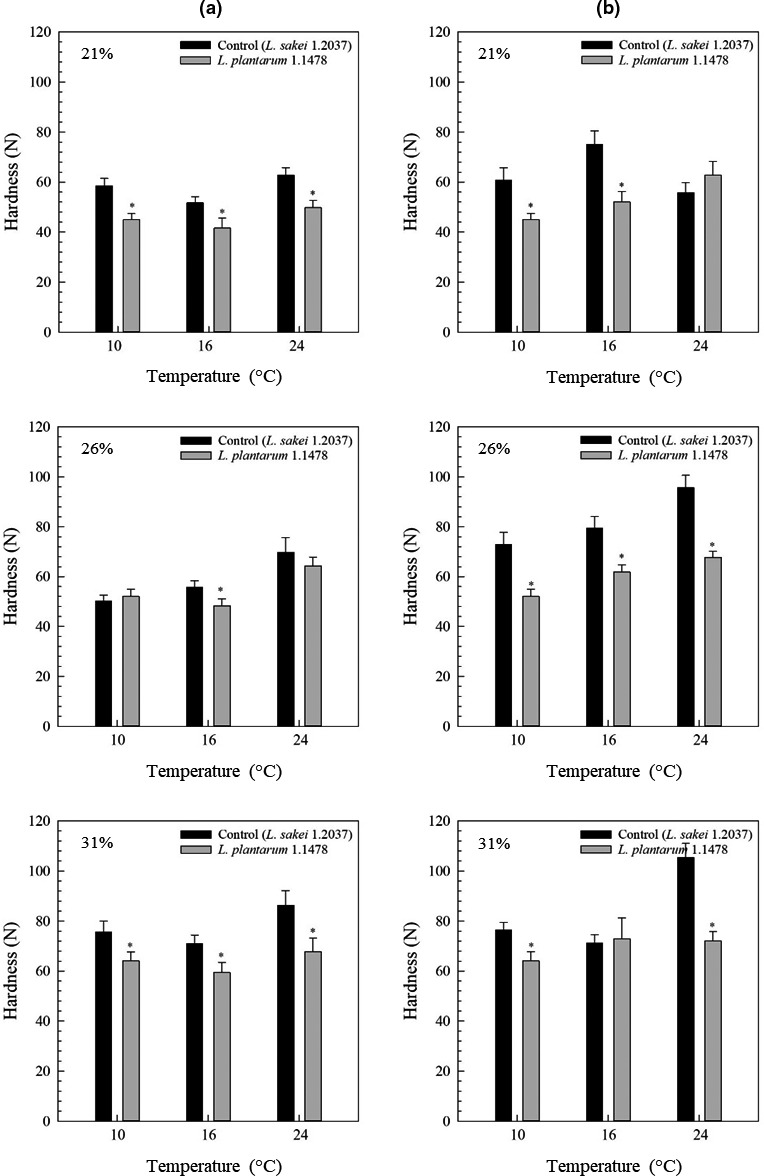
Hardness (*N*) of sausages produced with the non‐EPS‐forming strain *L. sakei* TMW 1.2037 (control; ~10^8^ CFU/g) or the HePS‐forming strain *L. plantarum* TMW 1.1478 (~10^8^ CFU/g) of the first (a) and second production (b; independent sausage production). Production was performed at different fermentation temperatures (10, 16, or 24°C) followed by a drying period at 14°C Above: Sausages reached 21% weight loss; center: Sausages reached 26% weight loss; below: Sausages reached 31% weight loss. An asterisk indicates significant differences to control samples (*p* < .05)

The in situ formed HePS by *L. plantarum* 1.1478 were qualitatively and semi‐quantitatively assessed using CLSM and a MATLAB approach following a method modified after Bosse et al. (2015). Figure 3 shows the EPS development over time in sausages containing the control strain *L. sakei* 1.2037 whereas Figure 4 presents the EPS development over time in sausages containing the HePS‐forming strain *L. plantarum* 1.1478. *L. plantarum* 1.1478 was able to form HePS during fermentation, while amounts stained in the control samples did not change remarkably over time. Since the biosynthesis of HePS is linked with the primary carbohydrate metabolism, EPS synthesis usually takes place during fermentation and partially at the stationary phase. This is in accordance with our findings, showing an increase in the quantities of HePS formed in the salami matrix within the first 48 hr of storage at 10, 16, or 24°C, respectively (Figure 4). While significant differences regarding EPS formation could be detected between the starter cultures, the different temperatures applied during the first 7 days of production (10, 16, and 24°C) had, according to the semi‐quantitative data interpretation (Table 1), a less pronounced influence on HePS‐production. Other studies reported on an enhanced EPS production when EPS‐forming strains were exposed to temperatures far below their optimum (van den Berg et al., 1995). The results found in the present study could be attributed to HePS being not homogenously distributed in the complex food matrix making it difficult to see differences between the EPS‐results gained at different temperatures. Latter one is additionally supported by the fact that usually very low amounts of HePS are formed. For instance, Hilbig, Gisder, et al. (2019) determined the EPS content in spreadable raw fermented sausages that have been produced with either the HoPS‐forming strains *L. sakei* TMW 1.411 or *L. curvatus* TMW 1.1928 or the HePS‐forming strain *L. plantarum* 1.1478 (fermentation at 24°C). Authors found high EPS‐concentrations in sausages containing one of the HoPS‐producing strains (0.46–1.03 g/kg) and significantly lower amounts in sausages containing *L. plantarum* 1.1478 (0.08–0.30 g/kg). HePS are known to cause structural changes at much lower concentrations than HoPS, which could explain the differences in weight loss even though the influence of temperature on HePS‐production could not be fully proven with the used semi‐quantitative approach. This is further supported by the results gained through the texture analysis (TPA), which revealed that sausages produced with the HePS‐forming strain were found to be significantly softer (*p* < .05) than the corresponding control samples as illustrated in Figure 5 (illustrated for 21%, 26%, and 31% weight loss). Furthermore, the gumminess, cohesiveness, and springiness of the products were influenced by both the present starter culture and the fermentation temperature used (Table 2), which could also be seen in the second, independent production (data not shown). These results are also reflected in the sensory evaluation (Figure S2), during which 20 panelists rated the sausages with HePS softer in terms of consistency. However, taste was not negatively affected.

**TABLE 1:** Results of the CLSM image analysis of sausages produced with the non‐EPS‐forming strain *L. sakei* TMW 1.2037 or the HePS‐forming strain *L. plantarum* TMW 1.1478 (~10^8^ CFU/g) of the first production

*T*_Fermentation_	Day	*L. sakei* 1.2037	*L. plantarum* 1.1478
	0	7.48 ± 2.87	7.48 ± 2.87
10°C	1	3.54^a^ ± 1.65	23.87^b^ ± 8.41
	2	4.02^a^ ± 1.16	20.87^b^ ± 11.57
	3	3.89^a^ ± 1.92	27.77^b^ ± 17.62
	~14	9.71^a^ ± 3.96	26.86^b^ ± 15.64
16°C	1	3.36^a^ ± 1.21	26.55^b^ ± 12.58
	2	6.29^a^ ± 3.24	32.25^b^ ± 12.70
	3	9.40^a^ ± 5.22	38.30^b^ ± 13.96
	~14	8.50^a^ ± 3.95	14.87^a^ ± 10.98
24°C	1	5.28^a^ ± 2.64	35.00^b^ ± 12.16
	2	6.91^a^ ± 3.16	25.14^b^ ± 7.46
	3	8.30^a^ ± 3.68	39.05^b^ ± 17.52
	~14	6.77^a^ ± 1.24	35.30^b^ ± 9.90

Note

Measurements were carried out over the fermentation and drying period until 31% weight loss was reached (~14 days); *n* ≥ 9 pictures. Values with different letters show significant differences (*p* < .05) within the row; ± is the standard deviation.

**TABLE 2:** Textural profile analysis parameters of sausages (31% weight loss) produced with the non‐EPS‐forming strain *L. sakei* TMW 1.2037 or the HePS‐forming strain *L. plantarum* TMW 1.1478 (~10^8^ CFU/g) and fermented at different temperatures; 1st production

Fermentation	*L. sakei* 1.2037	*L. plantarum* 1.1478
Gumminess (*N*)		
10°C	37.02^a,A^ ± 1.45	29.97^b,A^ ± 1.19
16°C	36.54^a,A^ ± 1.97	30.59^b,A^ ± 1.82
24°C	32.47^a,B^ ± 1.17	26.50^b,B^ ± 1.37
Cohesiveness (−)		
10°C	0.49^a,A^ ± 0.01	0.47^b,A^ ± 0.01
16°C	0.42^a,B^ ± 0.01	0.45^b,B^ ± 0.01
24°C	0.40^a,C^ ± 0.01	0.45^b,B^ ± 0.00
Springiness index (−)		
10°C	0.06^a,A^ ± 0.02	0.08^b,A^ ± 0.02
16°C	0.07^a,B^ ± 0.02	0.06^b,B^ ± 0.02
24°C	0.04^a,C^ ± 0.01	0.07^b,C^ ± 0.02

Note

Values with different capital letters show significant differences (*p* < .05) within the column whereas values with different lowercase letters indicate significant differences (*p* < .05) within the row; ± is the standard deviation.

To date, the functionality of EPS in various food matrices is yet not completely understood. Known is that monomer composition and ratio, degree of branching, charge density of EPS, as well as the actual food matrix, and extrinsic/intrinsic conditions during processing influence two phenomena that are of importance with regard to observed EPS functionalities: the polymer—solvent and the polymer—polymer interactions (van de Velde et al., 2015). In sausages with a high protein content such as salami (fat particles are surrounded by proteins), latter phenomena are more pronounced, especially at lower pH values since proteins are then often slightly positive charged. Since HePS are often negatively charged they readily associate with positively charged moieties on meat proteins thereby leading to structures (clusters—associative behavior) that influence the organoleptic properties of the final product. Depending on the type and amount of HePS formed this may cause different effects ranging from increased water binding to an increased spreadability as shown for teewurst (pronounced amount of HoPS), or to a harder or softer texture of final products. This is also supported by a study done by Ayala‐Hernandez et al. (2008) who proved that bacteria cells are able to bind to protein particles via EPS strands. Moreover, same authors reported that negatively charged EPS tend to aggregate with the milk protein phase as illustrated by CLSM. The formed clusters can then have different effects on the organoleptic properties of food products explaining why for instance Dertli et al. (2016) reported on harder and tougher raw fermented sausages (Sucuk – based on different raw material) while our results showed the exact opposite for salami. However, clear structure‐function relationships have yet to be established.

## 4 CONCLUSION

Using HePS‐forming starter cultures in raw fermented sausage manufacturing may lead to products with a softer texture, depending on the strain, matrix, and processing conditions used. Clear structure–function relationships still need to be developed taking not only the target matrix but also processing conditions into account. The present data indicate that one would need to consider the potential ability of starter cultures to produce EPS in situ in meat matrices thereby leading to desired or undesired properties highlighting the importance of a suitable starter culture selection in food processing.

## CONFLICT OF INTEREST

None of the (co‐) authors of this manuscript has a conflict of interest to declare.

## Supporting information

Figure S1‐S2

## Data Availability

The data that support the findings of this study are available from the corresponding author upon reasonable request.
