# Supplementary material for: Influence of fermentation temperature on in situ heteropolysaccharide formation (Lactobacillus plantarum TMW 1.1478) and texture properties of raw sausages
Source: Food Sci Nutr. 2021 Jan 23;9(3):1312–22. doi: 10.1002/fsn3.2054 (PMC7958580; doi:10.1002/fsn3.2054)
Supplement: Supplementary file 1 — Figure S1‐S2 [file FSN3-9-1312-s001.docx]

**Figure S1** Weight loss determination over time of raw fermented sausages that have been produced with the non-EPS-forming strain *L. sakei* TMW 1.2037 (control **A**; ~10^8^ CFU/g) or the HePS-forming strain *L. plantarum* TMW 1.1478 (**B**; ~10^8^ CFU/g) under different fermentation conditions 10, 16 or 24 °C.

**Figure S2** Sensory analysis of raw fermented sausages (31% weight loss) that have been produced using different fermentation conditions (10 °C, 16 °C or 24 °C) and either *L. sakei* 1.2037 (control; set as standard with a value of 5, which is indicated by the dotted line) or the HePS-forming strain *L. plantarum* 1.1478 of the first (**A**) and second (**B**) independent production. Significant differences between samples are indicated by an asterisk.

**S1**

**
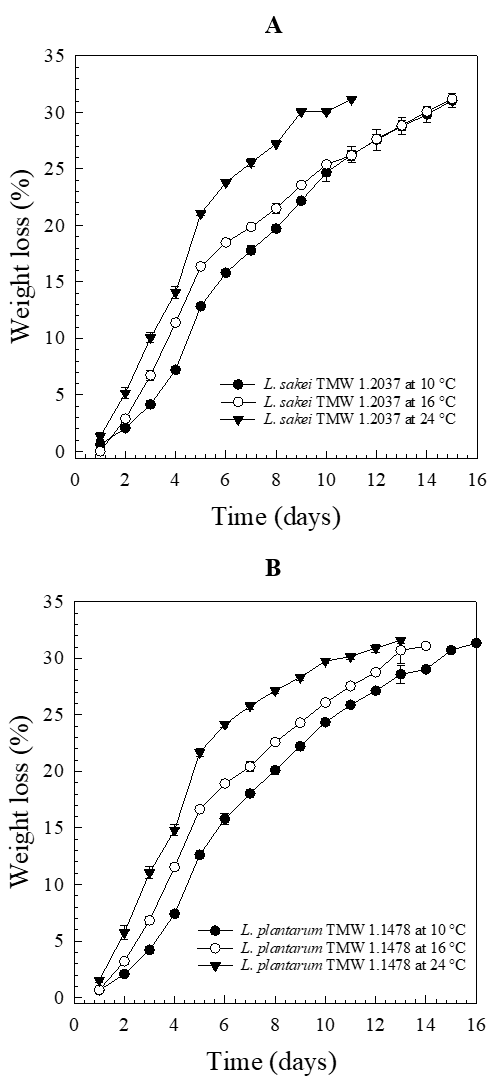
**

**S2**

**
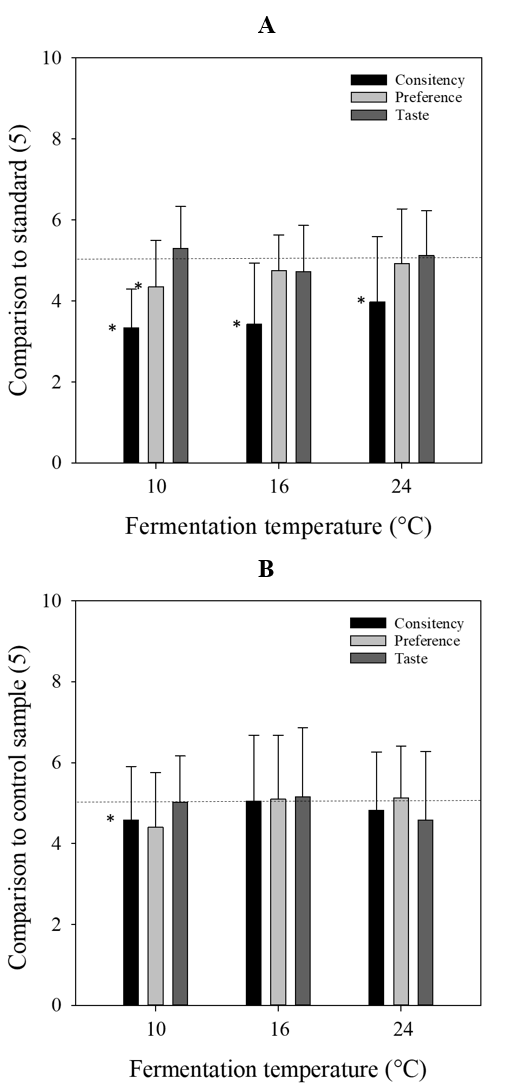
**
